# Podoconiosis today: challenges and opportunities

**DOI:** 10.1093/trstmh/try087

**Published:** 2018-08-22

**Authors:** Kebede Deribe

**Affiliations:** 1 Wellcome Trust Centre for Global Health Research, Brighton and Sussex Medical School, Brighton, UK; 2 School of Public Health, Addis Ababa University, Addis Ababa, Ethiopia

Twenty years ago, podoconiosis was hidden in the bookshelves and the suffering of people with the disease was a private matter that was not dealt with by the formal heath sector.^1^ For many health workers in endemic countries, podoconiosis was a mystifying dead end.^2^ However, with improved research, advocacy and demonstration projects, the disease came out of the ‘Dark Age’.^1^ Podoconiosis, which causes massive swelling of the lower leg, is caused by exposure to particles common in soils of volcanic origin^3^ and is second only to lymphatic filariasis as the leading cause of tropical lymphoedema.^4^ An estimated 4 million people live with podoconiosis globally in 32 potentially endemic countries.^5^ The control and elimination of podoconiosis rely on consistent footwear use from an early age and regular foot hygiene to avoid exposure to the type of soil responsible for the disease.^6^ For those already affected by the disease, the main strategy is hygiene-based management which includes foot hygiene, foot care, wound care, compression, exercises, elevation of the legs and treatment of acute infections.^7^

Today, podoconiosis is a disease of significant clinical and public health importance in several endemic countries. Over the last 15 years there has been remarkable progress in research, advocacy and implementation of podoconiosis inteventions.^1^ In 2011 the World Health Organization (WHO) recognized podoconiosis as one of the neglected tropical diseases (NTDs) under the category of ‘other tropical conditions’.^8^ Subsequently Ethiopia, Rwanda and Cameroon recognized podoconiosis as a priority NTD and included it in their long-term health plans.^6^ Mapping of podoconiosis has been completed in Cameroon, Ethiopia and Rwanda and evidence supporting the effectiveness of hygiene-based morbidity management has been established.^7^ As a consequence, morbidity management services have been integrated within the national health system in some countries such as Ethiopia.^9^ Progress over recent years has been impressive, but much remains to be done. There are several remaining challenges ahead to be addressed.

First, there is the challenge of recognition of podoconiosis as an important public health problem. The disease is one of the most disabling tropical diseases and is linked to significant stigma, discrimination and comorbid mental health problems.^10^ The disease has a significant impact in reducing the economic productivity of affected individuals and their families.^11^ All these justify the need for recognition of podoconiosis as a public health challenge and the development of a comprehensive and adequately resourced global health strategy towards elimination of podoconiosis. Advocacy is critical to increase awareness of the disease by decision makers. Identifying public figures as podoconiosis champions is important to achieve this goal. The use of different media outlets, including social media and newsletters, to increase public awareness is important. International podoconiosis initiative should be at the forefront of these efforts. The first international conference on podoconiosis will be held on 23 September 2018 in Addis Ababa, Ethiopia. The overall theme for the conference is ‘Research to Implementation: A Call for Global Action’. The conference should reach out to donors, policymakers and programme planners.

Second, in the constantly changing global health architecture, sustainable financing of podoconiosis research and intervention is a continuing challenge.^6^ Simple hygiene-based management for the treatment of podoconiosis has been shown to work. In a recent randomized controlled trial in Ethiopia, the intervention reduced the incidence of acute dermatolymphangioadenitis (one of the major sequela) by 20% in those treated.^7^ Scaling up these interventions is critical to alleviate the suffering of people with podoconiosis and improve their productivity and quality of life. Primary prevention interventions are also crucial. Empowering endemic communities to use footwear and undertake proper foot hygiene is important. Proven health messaging approaches and addressing practical and logistical challenges in accessing these interventions are needed.^12^ Sustainable financing is required to scale-up effective intervention. Donor funding for the scale-up intervention is needed, but to sustain the delivery of interventions it is critical to have domestic financing by endemic country governments.

Third, accurate data on the global burden and distribution of podoconiosis are critically required. As the former United Nations Secretary-General Kofi Annan said recently, ‘Without good data, we’re flying blind. If you can’t see it, you can’t solve it’.^13^ Investment in data generation for podoconiosis is a central point. Only 3 of the 32 potentially endemic countries have mapped the distribution of podoconiosis. Current global estimates rely on expert opinion. These data are barely enough to convince donors of the magnitude of the burden of podoconiosis for resource commitment or to provide ministries of health and policymakers with requisite data to plan interventions. Ethiopia is one of the countries in which podoconiosis has been mapped. Through mapping, 345 endemic districts were identified, an estimated 35 million people are at risk of the disease and there are an estimated 1.5 million cases.^14^ This evidence supported the initiation of a national podoconiosis program and its integration in the national health system.^15,16^

Fourth, progress in the innovation of new tools for podoconiosis is required, as few tools have been identified or developed for podoconiosis prevention and management. Right now is an exciting time when comprehensive analysis of genetic,^17^ epidemiological and environmental information combined with huge computational potential can be used to revolutionize podoconiosis research and innovation. Such analysis will have a two-pronged outcome: identifying the minerals responsible for podoconiosis and guiding the development of diagnostic and treatment tools by further increasing our understanding of the genetics, immunology and pathophysiology of podoconiosis. Innovations for the prevention of podoconiosis should also be explored. Tools for personal protection from exposure to soil should be developed. These tools should be acceptable, affordable and suit the specific context of people affected with podoconiosis and the environments they live in.

Fifth, translation of the evidence generated so far is needed. Over the last two decades the focus has been on podoconiosis research. Now it is time to translate the existing evidence into actual practice. Until recently, implementation of podoconiosis interventions was mainly by non-governmental organizations and often on a small scale. Thus there is a need for the governments of endemic countries to scale-up interventions. Health systems research focused on evaluation of the effectiveness of interventions and delivery mechanisms should also be conducted. Multidisciplinary and diverse research groups are needed to accelerate the elimination of podoconiosis.

In order to continue to foster progress on podoconiosis elimination, building on the gains so far is important to increase funding for research and implementation. Domestic financing of interventions and funding from the international community for research and development of new tools is important. Adoption of podoconiosis as an important NTD by the World Health Assembly is important to tackle the continuing challenge of neglect. Going forward, a global strategy needs to be put in place to tackle the issue of under implementation of podoconiosis programmes in endemic countries. This strategy, which could be developed by the WHO, should offer clearer guidance on proven interventions and implementation approaches. Along these lines, continued advocacy targeting bilateral donors and endemic country governments should emphasize greater recognition of podoconiosis as a disabling disease and a development challenge for endemic countries. Advocacy to include podoconiosis in the Global Burden of Disease (GBD)^18^ estimation should also be continued to generate robust global estimates that further advance the goal of elimination and serve as a tool for further evidence-based advocacy. The elimination of podoconiosis is an important goal and is achievable. Nonetheless, advancing this goal requires firm political commitment and sustainable policies. Endemic countries should have clear policies to address this important problem. These include solutions for universal access to affordable footwear, access to morbidity management services and well-designed community engagement activities.

Finally, history tells us that unless we keep momentum going, the gains achieved so far may be reversed. This is a clear reminder that we must take action to address the unmet needs of people with podoconiosis. Through targeted, evidence-based and cost-effective interventions, we can ensure podoconiosis patients and their families are afforded opportunities to realize their full potential. To achieve the elimination of podoconiosis, firm political will, policy formulation and operational and financial commitment are needed from donors, bilateral and multilateral organizations and, more importantly, endemic country governments.
